# Multiple Displacement Amplification as a Solution for Low Copy Number Plasmid Sequencing

**DOI:** 10.3389/fmicb.2021.617487

**Published:** 2021-02-11

**Authors:** Kuan Yao, Narjol González-Escalona, Maria Hoffmann

**Affiliations:** Division of Microbiology, Center for Food Safety and Applied Nutrition, Food and Drug Administration, College Park, MD, United States

**Keywords:** Salmonella, plasmid, sequencing, PacBio, multiplexing

## Abstract

Plasmids play a major role in bacterial adaptation to environmental stress and often contribute to antibiotic resistance and disease virulence. Although the complete sequence of each plasmid is essential for studying plasmid biology, most antibiotic resistance and virulence plasmids in *Salmonella* are present only in a low copy number, making extraction and sequencing difficult. Long read sequencing technologies require higher concentrations of DNA to provide optimal results. To resolve this problem, we assessed the sufficiency of multiple displacement amplification (MDA) for replicating *Salmonella* plasmid DNA to a satisfactory concentration for accurate sequencing and multiplexing. Nine *Salmonella enterica* isolates, representing nine different serovars carrying plasmids for which sequence data are already available at NCBI, were cultured and their plasmids isolated using an alkaline lysis extraction protocol. We then used the Phi29 polymerase to perform MDA, thereby obtaining enough plasmid DNA for long read sequencing. These amplified plasmids were multiplexed and sequenced on one single molecule, real-time (SMRT) cell with the Pacific Biosciences (Pacbio) Sequel sequencer. We were able to close all *Salmonella* plasmids (sizes ranged from 38 to 166 Kb) with sequencing coverage from 24 to 2,582X. This protocol, consisting of plasmid isolation, MDA, and multiplex sequencing, is an effective and fast method for closing high-molecular weight and low-copy-number plasmids. This high throughput protocol reduces the time and cost of plasmid closure.

## Introduction

*Salmonella enterica* is a Gram-negative foodborne pathogen associated with 1.2 million foodborne illnesses, 23,000 hospitalizations, and 450 deaths in the United States every year (Scallan et al., 2011). Due to selective pressures, prevalence of *Salmonella* isolates resistant to fluoroquinolones and third generation cephalosporin has been increasing in both clinical and agriculture settings (Karp et al., 2017). The National Antimicrobial Resistance Monitoring System (NARMS) has observed multidrug resistance *Salmonella* serotype doubling between the years 2011 and 2015 (CDC, 2018). The emergence of new isolates that are resistant to antibiotics, antimicrobials, sanitizers, and heavy metals complicates surveillance and prevention efforts worldwide. These forms of resistance also reduce the available methods for treating patients who contract resistant forms of salmonellosis.

Resistance can disseminate across serovars through mobile elements such as plasmids. Bacterial plasmids can carry genes conveying resistance to antibiotics, sanitizers, and/or heavy metals, as well as elements that enable toxin production, such as *ehxA* (Lindsey et al., 2009; Lorenz et al., 2016). In 2009, NARMS surveillance detected *Salmonella* Heidelberg isolates displaying extended-spectrum cephalosporin (ESC) resistance; these were found to be carrying plasmids encoding bla(CMY) beta-lactamase (Folster et al., 2012). More recent research has identified multidrug resistant *Salmonella* Infantis harboring *mcr-1* and *bla_CTZ-M-1_* genes in their plasmids (Carfora et al., 2018). The mobility of plasmids allows isolates to easily acquire new genes through conjugation, transposition, and site-specific recombination; once a plasmid is acquired, selection pressures may establish these traits throughout the population (Bennett, 2009; Liao et al., 2019).

Understanding the evolution of antimicrobial resistant (AMR) pathogens and the spread of mobile genetic elements which convey resistance, requires studying fully-closed sequences of plasmids. However, although whole genome sequencing (WGS) has become a reliable and comprehensive method for tracing the evolution of AMR pathogens (Hoffmann et al., 2014; Allard et al., 2016), plasmids have not received the same level of attention as chromosomal DNA. One of the obstacles to effectively obtain complete closed plasmid sequences is the usual multiple repetitive regions found on plasmids (e.g., insertion sequences) which make assembly with short reads difficult. Another obstacle is having enough initial DNA material to perform accurate sequencing using long read sequencing platforms, as larger plasmids typically exist in low copy numbers inside the cell.

One method for acquiring high molecular weight, low copy number plasmid DNA for sequencing on the Pacific Biosciences (Pacbio) sequencing platform was published by Hoffmann et al. (2017). Those authors obtained sufficient quantity of *Salmonella* plasmids using a modified plasmid mini preparation and then transformed the isolated plasmids by electroporation into *Escherichia coli* DH10Br in order to increase plasmid copy number. Then, the Qiagen Large-Construct kit™ (Qiagen, Gaithersburg, MD) was used to extract highly concentrated plasmid DNA suitable for sequencing with the Pacbio *RSII* (Pacific Biosciences, Menlo Park, CA, United States). This process allowed to completely close high molecular weight, low-copy number plasmids for *Salmonella*. However, any method that requires several steps of isolation and transformation into *E. coli*, consumes both laboratory resources and time, therefore a simpler, more efficient, and cost-effective method for closing high molecular weight, low copy number plasmids will be desirable.

In this report, we explore a different method for increasing the amount of plasmid DNA available for sequencing. We tested a method known as multiple displacement amplification (MDA; Cheung and Nelson, 1996), to replicate high molecular weight, low copy number plasmid DNA. Results obtained using MDA are very uniform across the target and have far less amplification bias compared to the traditional PCR-based whole genome amplification (WGA) technologies (Dean et al., 2002; Spits et al., 2006; Bleier et al., 2008). In contrast to PCR-based WGA technologies, Phi29 polymerase has 3'→5' exonuclease proofreading activity and maintains a much higher fidelity compared to Taq DNA polymerase during replication (Spits et al., 2006).

The process of MDA is a natural occurring method of replicating genetic material, used by some Gram-negative bacteria and some species of *Archaea* (del Solar et al., 1998). This simple mechanism is an exponential process, making it a very useful technique for amplifying circular DNA in the laboratory (Dean et al., 2002). Here, we show how switching from more labor-intensive methods of isolating and amplifying plasmid DNA to the extremely efficient MDA process will allow researchers to quickly acquire high molecular weight, low copy number plasmid DNA from foodborne pathogens in sufficient amounts, and of appropriate quality, for third generation sequencing techniques. By selecting bacteria whose plasmids have previously been sequenced, we will be able to determine the fidelity of amplification using MDA.

## Materials and Methods

### Bacterial Isolates

We selected nine *S. enterica* isolates representing nine different serovars. All isolates had been previously sequenced using Illumina short read and Pacific Bioscience long read sequence technology, analyzed, and their data deposited in the NCBI GenBank database. All isolates carried either one or two high molecular weight, low copy number plasmids, which ranged in size from 34,175 to 166,496 bp. The complete list of serovars and their metadata including accession numbers are listed in Table 1, and the complete reference sequences can be obtained from NCBI GenBank under https://www.ncbi.nlm.nih.gov/nucleotide/.

**Table 1:** Isolates selected for this study.

Strain#	Serovars	Location	Isolation source	Isolation date	PFGE XbaI pattern	Plasmid size (bp)	GenBank plasmid ID	Biosample #	Miseq data
CFSAN000006	Enteritidis	Mexico	poultry	n/a	n/a	59,336 (IncF^*^)	CP011395	SAMN01041085	SRR2544673
CFSAN001387	Tennessee	USA:GA	peanut butter	2007	JNXX01.0010	109,917 (IncFIB^*^)	CP014995	SAMN02261218	SRR965704
CFSAN001415	Weltefreden	USA:VA	tuna	2005	JQPX01.0092	81,966 (IncFII^*^)	CP014997	SAMN02568587	SRR3173804
CFSAN002064	Heidelberg	USA:WA	stool	2012	JF6X01.0122	37,692 (IncXI^*^)	CP005994	SAMN01933202	SRR924479
CFSAN002069	Heidelberg	USA:WA	chicken	2012	JF6X01.0122	37,679 (IncXI^*^); 110,363 (IncI^*^)	CP005389 CP005391	SAMN01933202	SRR924479
CFSAN007405	Typhimurium	USA:CA	turkey	2003	JPXX01.2132	132,146 (IncA/C^*^)	CP009410	SAMN02979242	SRR12480672
CFSAN007425	Newport	USA:MD	ground turkey	2002	JJPX01.0176	166,496 (IncA/C^*^)	CP009411	SAMN02737313	NZ_JAMQ00000000
CFSAN007426	Agona	USA:CO	ground turkey	2008	JABX01.0810	103,586 (IncA/C^*^)	CP009412	SAMN04218203	SRR3242350
CFSAN001297	Muenster	USA:NC	cow (fecal)	2001	n/a	34,175; 59,062 (IncI^*^)	CP019200 CP019199	SAMN01805332	SRR5819474

* Plasmid typing was carried out using PlasmidFinder-2.0 (Carattoli et al., 2014).

### Plasmid DNA Extraction

**Figure 1:**
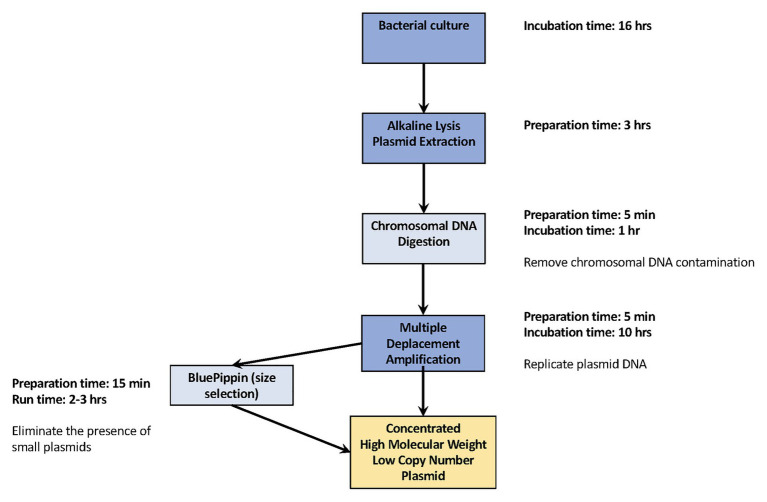
Workflow for replicating complete low-copy number plasmids. Our efficient workflow requires minimal hands-on preparation time, from overnight bacterial culture to generating concentrated high molecular weight plasmid DNA the following day. The dark blue squares are the essential steps for plasmid DNA replication. The light blue square provides the additional, recommended steps used in this study.

The workflow, beginning from bacterial culture and ending with producing concentrated, high molecular weight plasmid DNA, is summarized in Figure 1. The nine isolates were cultured separately in Luria Bertani broth (LB; Becton Dickinson, Franklin Lakes, NJ) overnight at 37°C, and plasmid DNA was extracted using an alkaline lysis extraction protocol previously reported by Hoffmann et al. (2017). Specifically, bacterial cells from overnight cultures were harvested by centrifugation at 6,000 *g* for 10 min at 4°C. The pellet for each isolate was resuspended in 350 μl of resuspension buffer containing: 1X Phosphate-buffered saline (ThermoFisher, Waltham, MA, United States), 40 mM Tris at pH8 (ThermoFisher, Waltham, MA, United States), 1 mM EDTA (ThermoFisher, Waltham, MA, United States), and 200 μg of RNase A (Qiagen, Hilden, Germany). Three hundred and fifty microliter of lysis buffer containing 0.05 N NaOH (ThermoFisher, Waltham, MA, United States) and 0.25% SDS (ThermoFisher, Waltham, MA, United States) was added to the bacterial mixture and mixed by gentle inversion. After 2-min incubation of the lysis buffer at 25°C, 500 μl of chilled 1.32 M potassium acetate buffer (ThermoFisher, Waltham, MA, United States) was mixed into the solution and incubated for an additional 5 min to neutralize the reaction. The supernatant was extracted after centrifugation at 20,800 *g* for 10 min at 4°C. An equal amount of phenol:chloroform:isoamyl alcohol solution (25:24:1; ThermoFisher, Waltham, MA, United States) was mixed with the supernatant until the solution had a homogenous milky appearance. This solution was centrifuged at 20,800 *g* for 8 min at 4°C. The clear top aqueous layer was taken and centrifuged at 20,800 *g* for 10 min at 4°C.

The supernatant containing plasmid DNA was transferred into a clean 1.5 ml tube. That DNA was precipitated by adding 0.1 volume of 3 M sodium acetate (ThermoFisher, Waltham, MA, United States) and 500 μl of 100% isopropanol (Sigma-Aldrich, St. Louis, MO, United States). The solution was mixed thoroughly by inversion and centrifuged at 20,800 *g* for 10 min at 4°C. The supernatant was discarded and the plasmid DNA pellet was washed twice with 1 ml of 70% ethanol (Sigma-Aldrich, St. Louis, MO, United States) and centrifuged at 20,800 *g* for 5 min. After air drying the plasmid pellet for 5 min, it was re-suspended in 12 μl ultra-pure DNase/RNase free distilled water (Thermo Fisher Scientific, MA, United States). Concentrations of plasmid DNA were measured using Qubit 3.0 fluorometer (Thermo Fisher Scientific, MA, United States) following the manufacturer’s protocol.

### Chromosomal DNA Digestion

Chromosomal DNA was removed from the isolated plasmids using Plasmid-Safe ATP-Dependent DNase (Epicenter, Madison, WI, United States) following the manufacturer’s preparation recommendations. First, excess enzymes were removed from the plasmid DNA solution through ethanol precipitation. About 0.5 μl of 3 M sodium acetate (ThermoFisher, Waltham, MA, United States) and 140 μl of cold 100% ethanol (Sigma-Aldrich, St. Louis, MO, United States) were added to the DNase treated plasmid DNA. The sample was then incubated at −20°C for 60 min and centrifuged at 20,800 *g* for 20 min at 4°C. After the supernatant was discarded, the pellet was washed twice with 1 ml of 70% ethanol and the plasmid DNA was re-dissolved in 20 μl ultra-pure DNase/RNase free distilled water (ThermoFisher, Waltham, MA, United States).

### Multiple Deplacement Amplification and DNA Size Selection

Once the plasmids had been extracted, we used the REPLI-g Mini kit (Qiagen, Hilden, Germany) to perform MDA (three replicates) for each set of plasmid DNA obtained.

We assessed the quality and the size of the plasmid DNA using the Fragment Analyzer DNF-464 High Sensitivity Large Fragment 50Kb analysis kit (Advanced Analytical Technologies, Inc., Ankeny, IA, United States). When the results of the Fragment Analyzer showed that an isolate carries both high molecular weight, low copy number plasmids along with low molecular weight, high copy number plasmids, size selection was performed. We used the BluePippin system (Sage Science, Inc., Beverly, MA, United States) to select for fragment sizes between 6 and 50 kb on a Free Marker S1 High-pass 6–10 Kb vs. three Agarose Gel Cassette (Sage Science, Inc., Beverly, MA, United States). All MDA products (size selected and non-size selected) were purified using Agencourt AMPure XP beads (Beckman Coulter), according to the manufacturer’s clean-up protocol. The concentrations of the purified MDA products were measured using Qubit 3.0 fluorometer (Thermo Fisher Scientific, MA, United States) following the manufacturer’s protocol.

### DNA Library Construction and Multiplex Sequencing

Amplified and purified plasmid DNA from nine isolates were used to construct multiplexed microbial SMRTbell libraries using the SMRTbell Template Prep Kit 1.0 (PacBio, Menlo Park, CA) according to the manufacturer’s protocol. Each isolate was ligated with a unique barcode using the SMRTbell Barcoded Adapter Complete Prep Kit-96 (PacBio, Menlo Park, CA). The barcoded adapters chosen for the study (listed in Table 2) were recommended by Pacific Biosciences for microbial multiplexing. These multiplexed SMRTbell libraries were then sequenced on a PacBio Sequel sequencer (PacBio, Menlo Park, CA) using PacBio Sequel V2.0 chemistry on one Sequel single molecule, real-time (SMRT) cell 1 M v2 (PacBio, Menlo Park, CA), with a 600-min collection time.

**Table 2:** Barcoded adapter names and sequences.

Isolate serovars	CFSAN#	Barcoded adapter name	Barcoded adapter Sequence
Enteritidis	CFSAN000006	BC1002	CTCACAGTCTGTGTGT
Tennessee	CFSAN001387	BC1016	ATACTATCTCTCTATG
Weltefreden	CFSAN001415	BC1032	CACTATCTCTAGTCTC
Heidelberg	CFSAN002064	BC1048	GAGTGTGAGTGCACAC
Heidelberg	CFSAN002069	BC1100	ACTACTGAGACATAGA
Typhimurium	CFSAN007405	BC1101	TATATCGCGTCGCTAT
Newport	CFSAN007425	BC1055	GATGAGATCTCGTGTG
Agona	CFSAN007426	BC1063	AGTGTGTCATGCGTGT
Muenster	CFSAN001297	BC1118	AGTATCATGTGTATCT

### Sequence Analysis

Raw sequencing data were demultipexed by running the Demultiplex Barcodes application with symmetric mode in SMRTLink v.5.1.0. (PacBio, Menlo Park, CA). The adapter sequences (Table 2) were trimmed and filtered out during the demultiplexing process. *De novo* assembly for each isolate was done using the PacBio hierarchical genome assembly process HGAP4.0 (Chin et al., 2013). We adjusted the target genome size in the assembly filter for each corresponding plasmid size while the other assembly parameters were set to Default. To check for genome closure, the overlapping regions were accessed using Gepard (Krumsiek et al., 2007). The plasmid genomes were aligned against their reference genomes obtained from GenBank. MAUVE aligner version 20150226 using progressive algorithm with default settings (Darling et al., 2004) was used to align each replicate against one other to assess the presence of variance.

## Results and Discussion

We developed a protocol for replicating low-copy number plasmid DNA in sufficient concentrations and quality to successfully close plasmid genomes. We then evaluated that protocol using three experimental replicates of nine *Salmonella* isolates to identify potential amplification biases that could occur during the MDA process. Our basic method of replicating low copy-number plasmid DNA requires as little as 3 h of hands-on time, providing only the essential steps for plasmid extraction and MDA (marked in dark blue in Figure 1) are performed. However, if you wish to acquire higher quality products for low copy number plasmids along with low molecular weight, our recommended additional steps, chromosomal DNA digestion and DNA size selection, requires only 20 more minuites hands-on time (marked in light blue in Figure 1). The higher the quality of the plasmid DNA, the more suitable it is for sequencing.

**Figure 2:**
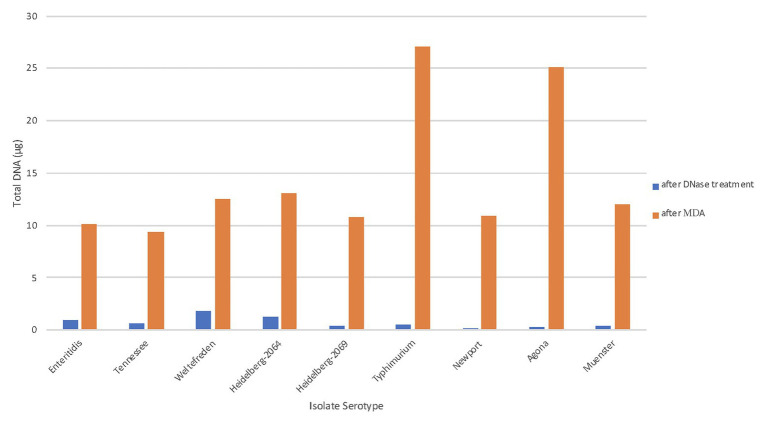
Total plasmid DNA (μg) after MDA. The total amounts of plasmid DNA in μg were measured at two different stages of the workflow. The blue bars represent the purified plasmid DNA after chromosomal digestion; the orange bar represent the total amplified DNA after MDA.

The size selection step is crucial for isolates that carry a larger plasmid along with a smaller plasmid, as size selection eliminates plasmids below 6 kb from the DNA to be amplified. Smaller plasmids are usually present in high copy number, therefore their DNA quantity will spike up during replication. That will cause problems during sequencing since most DNA will be amplified of the small plasmid DNA and only a small portion of the larger plasmid DNA of interest and therefore, the sequence data of the smaller plasmid will have a much higher coverage (data not shown). We assessed the quantity of plasmid DNA present after chromosomal DNA digestion treatment, and after performing the MDA (Figure 2). The average total plasmid DNA after chromosomal DNA digestion was 0.713 μg (range 0.3–1.7 μg), which includes also the amount of the smaller plasmid DNA (below 6 kb). After MDA, we found the total amount of purified plasmid DNA increased in average up to 14.5 μg (range 6–42 μg) which is an average increase of 20-fold and clearly shows that the new designed protocol is very effective. Hoffmann et al. (2017) obtained ~20 μg of high quality *Salmonella* plasmid DNA using their modified plasmid isolation protocol including transformation with *E. coli* DH10Br. The authors mentioned that at that time a minimum of 5 μg DNA was required for sequencing on the Pacbio *RS II*. Therefore, the goal of using MDA is to produce at least 5 μg DNA, which is successful as shown in Figure 2.

Having established that our method increased the quantity of plasmid DNA, we also assessed the quality of the DNA after amplification. Each amplification product was run on the Fragment Analyzer. The majority of plasmid DNA fragments obtained after MDA were above 30 kb, with average fragment sizes ranging from 35 to 45 kb. When the results of the Fragment Analyzer showed that an isolate carries both high molecular weight, low copy number plasmids along with low molecular weight, high copy number plasmids, it is important to perform size selection. The size selection is necessary in order to eliminate the low molecular weight, high copy number plasmids since they interfere with the results to achieve a high concentration for the high molecular weight, low copy number plasmids during MDA (experimental data not shown).

In order to assess whether any mutations or chimeras caused by the Phi29 DNA polymerase had been introduced during the MDA process, we performed three replicates of the MDA for all nine plasmid extraction products. Subsequently for each MDR product, a SMRTbell library was contructed and sequenced on the Pacbio Sequel sequencer. Although the total sequencing output, including all unbarcoded reads for each replicate, was 4.03, 2.39, and 1.69 Gb, after demultiplexing and sequencing read filtering, the actual barcoded sample output decreased to 1.2 Gb and 717 and 608 Mb. Using the PacBio hierarchical genome assembly process HGAP4.0, we performed *de novo* assemblies on 11 plasmid genomes from nine isolates for all three replicates (total = 33 assemblies). The assembled plasmid sizes and the corresponding coverage for each experimental replicate are displayed in Table 3. Our study included two isolates, *S.* Heidelberg CFSAN002069 and *S.* Muenster, that both carried two plasmids. For *S.* Muenster, two plasmid sizes were fairly close −34 and 59 kb; whereas *S.* Heidelberg CFSAN002069 carried one plasmid of 38 kb and another one of 110 kb. We obtained almost 2-fold coverage difference between the plasmids from *S*. Muenster (1,003 and 1779X), but found a 10-fold coverage difference between the *S.* Heidelberg plasmids (2,220 and 268X). When plasmids were close in size, there were less preferential amplification within the sample. Three (pCFSAN007405, pCFSAN007425, and pCFSAN007426) of the 11 plasmids from our study were previously included in the study from Hoffmann et al. (2017). At that time, the authors obtained for CFSAN007426 very similar sequence coverage and for the other two plasmids two to three times higher sequence coverage. However, at that time, the multiplexing protocol was not used instead each plasmid was sequenced seperately. In general, we generated very high coverage for each plasmid, therefore, it is possible to multiplex at least 10 times more plasmid DNA, and the cost for plasmid sequencing is reduced by multiplexing.

**Table 3:** Plasmid size and sequence coverage for each replicate.

	Replicate 1		Replicate 2		Replicate 3	
Isolate serovars	Plasmid size (bp)	Coverage	Plasmid size (bp)	Coverage	Plasmid size (bp)	Coverage
Enteritidis	59,372	1,177X	59,372	1,235X	59,372	499X
Tennessee	109,917	1,349X	109,917	461X	109,917	458X
Weltefreden	81,966	2011X	81,966	1,028X	81,967	553X
Heidelberg CFSAN002064	37,692	66X	37,692	24X	37,692	25X
Heidelberg CFSAN002069	110,363	268X	110,363	289X	110,364	145X
	37,697	2,220X	37,697	1931X	37,697	764X
Typhimurium	132,147	190X	132,149	223X	132,149	120X
Newport	166,506	417X	166,504	334X	166,505	323X
Agona	103,587	2,582X	103,588	787X	103,587	973X
Muenster	59,069	1779X	59,069	921X	59,069	661X
	34,175	1,003X	34,175	726X	34,175	939X

Two features of the sequencing performance may be worth elaborating: low sequencing output and the large variability in sequencing coverage. At the time (early 2018) when these experiments were performed, PacBio only offered Sequel V2.0 chemistry and Sequel SMRT cell 1 M v2. The microbial multiplexing barcode adaptor kit had not yet been released. Therefore, multiplexing protocol for greater than 10 kb libraries had not yet been fully optimized. Given these conditions, our actual sequencing output was less than optimal (4 Gb at best). After read filtering for barcode adapters, most of the unbarcoded reads and low quality reads were excluded. However, this left us with significantly less (~70%) data for *de novo* assembly. In the case of *S.* Heidelberg CFSAN002064, the sequencing coverage was decreased by (a) low total sequencing output, and (b) read filtering. Since then, PacBio had made significant upgrade in their chemistry and barcoded adaptor kit. New Sequel V3.0 chemistry can provide much higher sequencing output (~8 Gb) and better barcode recognition (Pacific-Bioscience, 2019).

After analyzing the sequence data, the results were compared with the published sequences for each plasmid that had previously been archived in the NCBI GenBank. In contrast to PCR-based WGA technologies, Phi29 polymerase has 3'→5' exonuclease proofreading activity and maintains up to 1,000-fold higher fidelity compared to Taq DNA polymerase during replication. In order to detect sequencing single nucleotide polymorphisms (SNPs) or variants, we aligned the assembled genomes against each other using Mauve (Table 4). Out of the 33 assembled plasmids, 24 did not have any SNP differences and eight plasmids had only one SNP compared to the reference genomes. Only in *S.* Newport that carried the largest plasmid, we detected in one replicate five SNPs. The SNPs between three replicates from one plasmid were never detected on the same base position within the plasmid. In particular, we identified eight instances of addition or deletion occurred in homopolymer regions. There were three instances of a single base insertion/deletion, and a single instance of two base-pair deletion. No substitutions were observed. Interestingly, variants appeared disproportionately in AT-rich regions. There were six variants in poly-A or poly-T regions, but only two variants observed in the poly-G region. Most of the variants, we observed consisted of a deletion or an insertion in a homopolymer; these kinds of errors are known to occur as an artifact of sequencing rather than representing an artifact of the MDA process.

**Table 4:** Number of variants observed in genome assemblies between the three replicates.

Isolate name	# of variance replicate 1	# of variance replicate 2	# of variance replicate 3
Enteritidis	–	–	–
Tennessee	–	–	–
Weltefreden	–	–	add 1A in homopolymer stretch
Heidelberg CFSAN002064	–	–	–
Heidelberg CFSAN002069	– –	– –	add 1 T in homopolymer stretch –
Typhimurium	del 1A in homopolymer stretch	add A	add 1A to homopolymer stretch
Newport	del T	del AC, add T, del 1G in homopolymer stretch, del 1A in homopolymer stretch	–
Agona	–	del 1G in homopolymer stretch	add 1A to homopolymer stretch
Muenster	– –	– –	– –

“Add” indicates addition of a nucleotide, “Del” indicates deletion; no substitution was observed.

## Conclusion

Our experiments demonstrate that MDA provides a more efficient way to prepare low copy number plasmids for sequencing: it requires much less time and effort than using electroporation to insert the plasmid inside *E. coli* in order to increase plasmid numbers (Hoffmann et al., 2017), and our process minimizes amplification bias allowing closure of high molecular weight plasmids. By using a single MDA reaction after plasmid isolation, we were able to produce adequate quantities of high quality plasmid DNA for long read sequencing. We successfully closed 11 plasmids ranging between 37 and 166 kb, isolated from nine different *Salmonella* serovars. Using MDA, the amount of plasmid DNA produced can be increased 20-fold and enables researchers to conveniently replicate large quantities of low copy number, high molecular weight plasmid DNA, producing material suitable for sequencing on the PacBio Sequel. Utilizing the multiplexing protocol further maximized the throughput and reduced the cost for plasmid closure. Our method may also be applied to manipulating any other circular DNA, such as mitochondrial DNA or chloroplast DNA.

## Data Availability Statement

The original contributions presented in the study are included in the article/Supplementary Material, further inquiries can be directed to the corresponding author.

## Author Contributions

All authors played an integral part of project conception. Each author has read and approved the final version of the manuscript. Specifically, NG-E and MH conceived and designed the experiments. KY performed the experiments. KY and MH analyzed the data and wrote the manuscript.

### Conflict of Interest

The authors declare that the research was conducted in the absence of any commercial or financial relationships that could be construed as a potential conflict of interest.

## Supplementary Material

Supplementary Figure 1 Fragment size on amplified plasmid DNA. The fragment sizes of plasmid DNA acquired after MDA were measured using Fragment Analyzer. The average fragment sizes (in bp) are shown on the top. The amplified products ranged between 35 and 45 kb.
