# Supplementary figures and images for: Multiple Displacement Amplification as a Solution for Low Copy Number Plasmid Sequencing
Source: Front Microbiol. 2021 Feb 11;12:617487. doi: 10.3389/fmicb.2021.617487 (PMC7904871; doi:10.3389/fmicb.2021.617487)

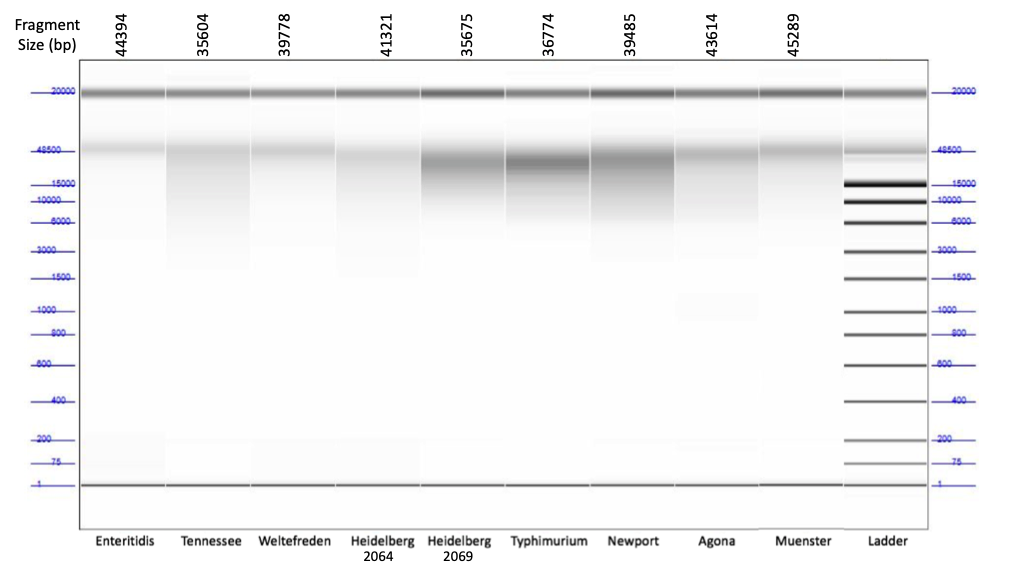

Supplement: Supplementary Figure 1 — Fragment size on amplified plasmid DNA. The fragment sizes of plasmid DNA acquired after MDA were measured using Fragment Analyzer. The average fragment sizes (in bp) are shown on the top. The amplified products ranged between 35 and 45 kb. [file Image_1.tif]
